# Are Community Acquired Respiratory Viral Infections an Underestimated Burden in Hematology Patients?

**DOI:** 10.3390/microorganisms7110521

**Published:** 2019-11-02

**Authors:** Cristian-Marian Popescu, Aurora Livia Ursache, Gavriela Feketea, Corina Bocsan, Laura Jimbu, Oana Mesaros, Michael Edwards, Hongwei Wang, Iulia Berceanu, Alexandra Neaga, Mihnea Zdrenghea

**Affiliations:** 1 Department of Hematology, Iuliu Hatieganu University of Medicine and Pharmacy, 8 Babes str., 400012 Cluj-Napoca, Romania; Fechetea.Gabriela@umfcluj.ro (G.F.); jimbulaura@yahoo.com (L.J.); mesaros.oana@umfcluj.ro (O.M.); Neaga.Alexandra@umfcluj.ro (A.N.); 2 The Faculty of Veterinary Medicine, University of Agricultural Sciences and Veterinary Medicine, Calea Mănăștur 3-5, 400372 Cluj-Napoca, Romania; aura_ursache@yahoo.com; 3 Department of Pharmacology, Toxicology and Clinical Pharmacology, Iuliu Haţieganu University of Medicine and Pharmacy, 400337 Cluj-Napoca, Romania; bocsan.corina@umfcluj.ro; 4 National Heart and Lung Institute, St Mary’s Campus, Imperial College London, London W2 1PG, UK; michael.edwards@imperial.ac.uk; 5 Medical Research Council (MRC) and Asthma UK Centre in Allergic Mechanisms of Asthma, London W2 1PG, UK; 6 School of Medicine, Nanjing University, 22 Hankou Road, Nanjing 210093, China; hwang@nju.edu.cn; 7 Department of Hematology, Ion Chiricuta Oncology Institute, 34-36 Republicii Street, 400015 Cluj-Napoca, Romania; iuliaberceanu@yahoo.com

**Keywords:** respiratory viral infections, community respiratory viruses, hematological malignancy, stem cell transplantation, intensive chemotherapy

## Abstract

Despite a plethora of studies demonstrating significant morbidity and mortality due to community-acquired respiratory viral (CRV) infections in intensively treated hematology patients, and despite the availability of evidence-based guidelines for the diagnosis and management of respiratory viral infections in this setting, there is no uniform inclusion of respiratory viral infection management in the clinical hematology routine. Nevertheless, timely diagnosis and systematic management of CRV infections in intensively treated hematology patients has a demonstrated potential to significantly improve outcome. We have briefly summarized the recently published data on CRV infection epidemiology, as well as guidelines on the diagnosis and management of CRV infections in patients intensively treated for hematological malignancies. We have also assessed available treatment options, as well as mentioned novel agents currently in development.

## 1. Introduction

Community respiratory viruses (CRVs) are becoming increasingly recognized as a significant cause of morbidity and mortality among patients with hematologic malignancies. While epidemiological studies on bacterial infections in such patients are feasible, with culture methodologies proving to be a reliable and accessible method of investigation, the limited availability of highly sensitive and specific virological diagnostic tests have made the corresponding data much more difficult to obtain. The advent of nucleic-acid-based testing has made it possible to not only obtain positive diagnoses for respiratory viral infections accurately, reliably, and quickly, but to do so without having to rely on specialized centers for viral cultures.

Outcomes for intensively treated hematology patients have improved consistently throughout the years, and while the development of newer, more effective treatment regimens was responsible for initial leaps forward in terms of survival, much of the progress made in recent decades can be attributed to an improvement in supportive measures, including antibiotic, antifungal, and, when applicable, antiviral treatment, additively leading to a better tolerance of aggressive regimens and improved long-term survival.

Thus, in the last two decades, the interest in CRVs has increased and a number of papers have explored their role in respiratory disease in immunodeficient patients. It is of note that important respiratory viruses associated with the above were found to be not only common respiratory viruses, such as influenza, parainfluenza, respiratory syncytial virus (RSV), adenoviruses, rhinoviruses (HRV), and coronaviruses, but also newly identified viruses, such as human metapneumoviruses (HMPV) [1,2,3], bocavirus (HBoV) [4,5,6], and new strains of coronavirus [7]. Most of these findings applied to specific population groups, such as children, the elderly, transplant patients undergoing immunosuppressive treatment, and patients with hematological malignancies (Table 1).

Diseases caused by these viruses vary from self-limiting upper respiratory tract infections (URTIs) to life-threatening lower respiratory tract infections (LRTIs), as well as occasional disseminated disease. Progression from the former to the latter, with clinical and radiological signs of pneumonia, depends both on the intrinsic virulence of the specific CRVs and on patient-related factors such as age, underlying illness, type, and degree and duration of immune deficiency [8]. Pneumonia following URTIs may be primarily viral, bacterial, fungal, or mixed, with certain CRVs, such as parainfluenza, being more frequently associated with co-pathogens [9].

Respiratory tract infections exhibit the seasonality shared by most CRVs, with community outbreaks of RSV infections typically occurring during late autumn, winter, and early spring, followed by outbreaks of HMPV. Influenza outbreaks generally take place during winter in temperate climates (but may occur throughout the year in tropical areas), while parainfluenza virus infections may appear throughout the year, with outbreaks mainly occurring in the spring, summer, and autumn [9]. Several theories have been proposed to explain this seasonality: increased crowding, which facilitates transmission, happens during the cold seasons; lower temperatures may increase the stability of virions outside the body; chilling may increase host susceptibility and lower temperatures or host chilling may activate dormant virions; and vitamin D deficiency is more prevalent in winter due to lower sun exposure [10].

To better define the impact of CRV infections in hematology patients, a working group of the Fourth European Conference on Infections in Leukemia (ECIL-4) reviewed the literature on community respiratory viruses and, in January 2013, made recommendations according to the Infectious Diseases Society of America grading system [8].

Diagnosis of CRV infection is made using specimens from nasopharyngeal aspirates, nasopharyngeal swabs, tracheal aspirates, and bronchoalveolar lavage. The main laboratory tests used are nucleic acid amplification testing (NAT), direct antigen testing (DAD), and virus isolation by cell culture (VIC). NAT is often preferred, having a higher sensitivity and shorter turn-around time (<24 h), as well as the possibility of multiplexing [11,12,13,14]. Because NAT is capable of detecting viral nucleic acids in asymptomatic patients [12], it becomes important to distinguish between CRV infection and CRV infectious disease; to this effect, the ECIL-4 group adapted the case definition proposed by the European Centre for Disease Prevention and Control for influenza virus for other CRVs in patients with leukemia and hematopoietic stem cell transplantation (HSCT) (Table 2).

We have tried to update the ECIL-4 review with data from newer studies, including data on influenza infections. We have also tried to add some insight into some of the novel therapeutic agents currently in use or in the development pipeline. In line with ECIL-4’s findings, the trend of retrospective, observational studies representing the majority of publications has continued.

## 2. Evidence of CRV-Infection-Related Morbidity and Mortality in Hematology Patients

Human influenza virus (flu), a member of the Orthomyxoviridae family, is perhaps the most thoroughly studied of all CRVs. The virus has a spherical (and sometimes filamentous) structure, 80 to 120 nm in diameter, with a segmented, negative-strand RNA genome complexed with a nucleoprotein to form a nucleocapsid with helical symmetry. The nucleocapsid is enclosed in a lipid envelope with two surface glycoproteins, a hemagglutinin and a neuraminidase, with roles in both pathogenesis and classification of viruses in subtypes [18].

The importance of the flu virus as a human pathogen has been well documented through history: historically, the first, generally agreed-upon influenza pandemic occurred in 1580 (originating in Asia); it was followed, in the coming centuries, by pandemics in 1729 (Russia), 1781–1782 (China), 1830–1833 (China), 1898–1900 (Russia), 1918–1920 (USA), 1957–1958 (Yunnan Province, China), 1968–1970 (Hong Kong), and 2009–2010 (Mexico), each subsequent pandemic with increasingly accelerated spread due to advances in transportation infrastructure and globalization [19,20]. The 1918–1919 pandemic alone was responsible for an estimated death toll of 50 million (or higher) [21]. The Center for Disease Control and Prevention (CDC) has made yearly estimates of flu deaths in the US for the past several decades; this information was last updated in 2010 with data from 1976–2007. According to their estimates, the number of influenza-related deaths varies substantially depending on year, virus type/subtype, and age group. More precisely, the estimated number of flu-related deaths due to respiratory and/or circulatory causes has ranged from 3349 (in the 1986–1987 season) to 48,614 (2003–2004 season), with an average of 23,607 annual flu-related deaths, of which persons of age 65 or older account for approximately 90%. Seasons with prominent influenza A (H3N2) circulation have been shown to have 2.7 times more deaths than seasons where influenza A (H3N2) was not prominent [22]. Similar estimates on a continental scale are difficult to obtain in Europe due to the large disparity between countries in terms of diagnosis and surveillance. However, national estimates are available, such as the systematic literature review performed by Buchholz et al. on influenza pandemic deaths in Germany from 1918 to 2009; they estimated the influenza-related mortality to range from 426,000 (1918–1919) to 350 (2009) [23].

Several risk factors have been associated with poor outcome in influenza infection: lymphopenia [24,25,26,27], myeloablative versus non-myeloablative conditioning regimens [28], low serum albumin levels [29], and delayed antiviral treatment [29,30,31]. The risks and benefits of corticosteroid use are debatable, as discussed below.

The 2009 pandemic had 18,631 laboratory-confirmed pandemic deaths reported by the World Health Organization. However, the total mortality burden was estimated to be 10 to 15 times higher, with a surprisingly high (62–82%) percentage of deaths attributed to persons under 65 years of age [32,33]. Considering that the estimated cumulative worldwide incidence was 24% (1.63 billion people) [34], the above figures amount to a 0.01% to 0.016% global mortality. While no conclusive large-scale studies are available to estimate the pandemic’s global impact on hematological patients in terms of mortality, a number of single-center studies showed a variety of results. Mortality appears to have been similar to the general population’s [35,36] with a greater duration of hospitalization and antiviral treatment for hematology patients [36]. However, data from two European prospective surveys showed H1N1-pandemic-related mortality in HSCT patients to be 6.3%, though the design of both studies probably identified the more severe cases [26]. Another study, comparing pandemic to post-pandemic influenza outbreaks in hematology patients showed a higher progression to pneumonia in the H1N1 pandemic group; there was, however, no difference in mortality [29]. Likewise, a study directly comparing 2009 AH1N1 pandemic to seasonal A and B influenza infections in HSCT recipients showed an increase in progression to LRTI and hypoxemia requiring mechanical ventilation in patients infected with the pandemic strain, with no significant differences in mortality; prompt antiviral therapy and high-dose steroids were shown to reduce the need for mechanical ventilation [30]. Conversely, it was shown that not only did corticosteroids not reduce progression to LRTI or mechanical ventilation, but that high-dose corticosteroid treatment (≥1 mg/kg) prolonged viral shedding [37]. Likewise, Martin-Loeches et al. showed that early use of corticosteroids in patients with AH1N1 2009 pandemic influenza who were admitted to the ICU was associated with increased risk of superinfections [38].

Human respiratory syncytial virus (RSV), belonging to the Paramyxoviridae family, contains a continuous, single-stranded negative-sense RNA genome which is encapsulated by a nucleoprotein, phosphoprotein, and RNA-dependent RNA polymerase to form the ribonucleoprotein complex; the virus possesses three membrane proteins: the receptor attachment glycoprotein, the fusion protein, and a short hydrophobic protein [39]. RSV infections occur year-round, but peak during the cold season; they mostly affect young children, who develop URTI disease (rhinitis, laryngitis, sinusitis), and neonates, at risk for LRTI disease (bronchiolitis, pneumonia), with a meta-analysis revealing a conservative estimation of 33.8 million RSV-related LRTIs worldwide in children younger than 5 years (roughly 22% of all LRTIs in young children), resulting in 66,000–199,000 deaths in 2005 [40].

Patients with hematological malignancies and/or HSCT have an increased risk of community-acquired, household, and nosocomial transmission [41,42]. RSV infections occur in 0.3–2.2% of pediatric patients with acute myeloid leukemia [43], 14% of children with acute lymphoblastic leukemia [44], and 8.7% of children with various malignancies, as shown by a Brazilian group [45]. Studies on adult populations with hematological malignancies and/or HSCT have revealed incidences ranging from 1% to 31% [25,46,47,48,49,50,51] (Table 3). Progression to LRTI disease was reported in 38% of leukemia and HSCT patients, with an average mortality rate of 32% [52,53]. Patients with HSCT evaluated prospectively during a single influenza season in Switzerland showed RSV infection in 7.95% of swabs (21/264 swabs, from a total of 193 patients) [54]. Long-term (with a median duration of 80 days and a range of 35–334 days) shedding of RSV was demonstrated in HSCT recipients in Germany, emphasizing the need for infection control and nosocomial transmission prevention measures [55].

Risk factors for progression to LRTI in RSV infections have primarily been found to be host-related, with no significant difference in outcome between RSV serotypes A and B [56,57]. A comprehensive systematic review conducted in 2015 identified eight risk factors significantly associated with RSV LRTI in children: prematurity, low birth weight, male sex, having siblings, maternal smoking, history of atopy, lack of breastfeeding, and crowding [58]. In HSCT recipients, smoking history, conditioning with high-dose total body irradiation, and an absolute lymphocyte count ≤100/mm^3^ at the time of URTI onset were significantly associated with disease progression [57]. As with influenza, corticosteroid therapy at symptom onset of URTI was shown by Damlaj et al. to be significantly associated with a higher progression to LRTI (risk ratio (RR) = 2.49 (1.21–5.13; *p* = 0.016)), hospital admission (RR = 2.05 (1.24–3.37; *p* = 0.005)), or ICU admission (RR = 2.91 (1.89-5.01; *p* = 0.002)), but not with mortality (*p* = 0.26) [59]. In a 42 month retrospective observational study from Michigan investigating patients admitted with RSV infection, no significant increase in morbidity or mortality was identified in patients with HSCT or solid organ transplant versus immune-competent patients, mortality being higher, however, for patients over 60 years or with lymphopenia on admission [60]. Waghmare et al. identified RSV RNA detection in plasma or serum as a potential marker for poor outcome in HSCT recipients with RSV LRTI [61].

In order to facilitate the identification of at-risk HSCT candidates, an immunodeficiency scoring index (ISI) for RSV was developed, measuring six factors: neutropenia <500 neutrophils/mL, lymphopenia <200 lymphocytes/mL, age >40 years old, graft-versus-host disease, corticosteroid use, myeloablative chemotherapy, and time from HSCT. Based on the total score, HSCT recipients with URTI are stratified by the ISI into low-risk (score 0–2), medium-risk (score 3–6), and high-risk (score 7–12) categories. The ISI was verified in a subsequent study, with high score (≥8) predicting progression to LRTI with a positive predictive value of >80% for URTI caused by RSV, influenza, parainfluenza, and adenovirus, but without being predictive for coronavirus and rhinovirus [62].

Human metapneumovirus (HMPV) is a negative-sense, non-segmented, single-stranded RNA virus belonging to the Paramyxoviridae family, identified in 2001 by a Dutch group [63,64]. It shares many similarities with RSV and has been increasingly recognized as a leading cause of RTIs in both children and adults. Since its discovery, seroprevalence studies across the globe have indicated that primary infection happens before the age of 5 and virtually all children are infected by the age of 10 [65,66,67,68], with reinfection occurring throughout life [69]. HMPV demonstrates remarkable robustness through a variety of mechanisms, the description of which are beyond the scope of this article, but which have been thoroughly investigated elsewhere [70,71,72,73,74,75].

Among immunocompetent hosts, HMPV accounts for 2% to 7% of CRV infections; a study done in Nashville testing nasal-wash specimens obtained over a 25 year period from otherwise healthy children presenting with acute LRTI identified HMPV RNA in 20% of viable specimens [76]. In patients with hematological malignancies or HSCT recipients, HMPV detection rates range from 2.5% to 9% in the first 2 years after transplantation [77,78,79,80]. A systematic review including 17 studies, published in 2016 by Shah et al., showed an incidence of HMPV infections of 5% (with a reported range of 0% to 40%) in hematological malignancy and HSCT patients [81]. Despite being typically self-limiting when infecting the general population, there have been reported cases of severe disease and fatal outcomes, especially among HSCT patients [82,83,84], although frequent coinfection makes mortality directly attributable to HMPV difficult to ascertain. Among immunocompetent children, prematurity, female sex, and genotype B infection were associated with severe HMPV disease [85], while for cancer patients, it has been shown that hypoxia, nosocomially acquired HMPV infection, and the presence of hematological malignancy represent risk factors for progression to LRTI [86]. Notably, in the study mentioned above, risk factors traditionally associated with poor outcomes in other respiratory viruses, such as older age, smoking history, or corticosteroid therapy, were not shown to negatively influence outcome in HMPV infection [86].

Human rhinoviruses (HRVs), a group of positive-sense, single-stranded RNA viruses belonging to the Picornaviridae family, circulate throughout the year and are the most common cause of URTIDs, having been demonstrated to be responsible for 52.5% to 79.68% of common colds [87,88,89]. While largely benign in immunocompetent patients, their role in the morbidity and mortality of at-risk populations has only come to attention only in recent years. In children with hematological malignancies and/or HSCT, HRV was detected in 23.1% to to 62% of URTIDs [45,90,91,92] and 65% of LRTIDs [90]. Notably, one study from Toronto identified HRV in 2% of documented RTIs in pediatric HSCT recipients [93]. In adults with HSCT, HRV maintains its top position insofar as frequency is concerned, reaching a cumulative incidence of 22.3% by day 100 post-transplant [7]. It was identified as the second most frequent cause of idiopathic pneumonia syndrome in HSCT patients, representing 12% of detected pathogens [94]. In a study on neutropenic, non-HSCT patients with hematological malignancies followed over 26 months, HRV was detected in 11/144 patients with nasopharyngeal aspirate samples (7.6%), five of whom were asymptomatic [95]. A 3 year retrospective study from New York showed that 39.6% of HSCT patients with documented HRV infection had proven pneumonia, and of those, 60% were demonstrated coinfections [96]. Low monocyte count, oxygen requirement at diagnosis, and corticosteroid use ≥1 mg/kg were identified as risk factors for overall mortality in HRV infections [97].

Human enteroviruses (HEV), members of the Picornaviridae family, encompass more than 70 serotypes. In a retrospective study of patients with hematological malignancies diagnosed with respiratory infections with HEV or HRV, LRTI was preset at onset in 33% of cases, with progression from URTI to LRTI occurring in 6% of cases; direct impact on mortality was uncertain due to the frequency of copathogens [98]. On the other hand, case reports have described severe disease with fatal outcomes in HSCT patients with HEV as the sole identifiable pathogen [99].

The importance of HEV infections among hematology patients may have been understated, even more so than other respiratory viruses. Enterovirus D68 (EV-D68), first identified in 1962 [100], has since been associated with small outbreaks worldwide, affecting predominantly children, with severity ranging from mild to isolated fatalities [101,102,103,104], including one case through aseptic meningomyeloencephalitis [105]. In a study by Waghmare et al., 8 out of 21 presumed EV-D68 cases (38%) were patients with hematological malignancies [106]. Outcomes were consistent with those reported in infections with other respiratory viruses (though the small number of patients makes the results difficult to extrapolate). Notably, the same study showed that 10% (11/113) of patients who had tested positive for HRV infection were, in fact, infected with EV-D68, as was proven after sequencing [106].

Another enterovirus, EV109, identified in 2010 from nose and throat swab samples collected from a pediatric cohort study of influenza-like illness in Nicaragua [107], was found in 1/175 samples from HSCT recipients (as well as 2/974 samples from infants 1 to 24 months old) in Italy; the positive sample from the HSCT was taken 180 days after transplantation [108]. The relatively small group of hematology patients investigated for EV109, as with EV-D68, makes their impact on this population group difficult to fully appreciate, warranting further and larger studies.

Human coronaviruses (HCoV), belonging to the Coronaviridae family, are large, enveloped, positive-strand RNA viruses, the genome of which is packed inside a helical capsid and surrounded by an envelope with three associated proteins: the membrane protein and the envelope protein (both involved in viral assembly), and the spike protein (which facilitates viral entry into host cells) [109]. Coronaviruses circulate throughout the year, with a slight predominance during the winter. Although largely benign, presumably responsible for 10%to 30% of common colds [8], their ability for animal to human and rapid interhuman transmission have made them one of the most dangerous respiratory pathogens of the new century, as exemplified by the severe respiratory syndrome coronavirus (SARS-CoV, with 8422 people infected worldwide within seven months of disease emergence, with 916 (10.8%) fatalities [110]) and Middle East respiratory syndrome coronavirus (MERS-CoV, with 2206 people infected and 787 (35.67%) fatalities as of April 2018 [111]).

In HSCT patients, HCoV reached a cumulative incidence of 11.1% on day 100 post-transplant [7]. Another study identified HCoV in 6.8% of samples positive for any respiratory virus gathered over a three year period, with 86.8% of patients in whom HCoV was identified being HSCT or hematological malignancy patients [112]. Pinana et al. found a total of 21 HCoV infectious episodes in 18/79 (23%) HSCT recipients, 7 of which progressed to LRTI [113]. While LRTI is traditionally considered rare compared to other respiratory viruses, with progression to pneumonia with severe outcomes having been described in case reports [114,115], recent data suggest that mortality from HCoV LRTI may be higher than originally thought, and similar to that seen with RSV, influenza, and HPiV infections; as Ogimi et al. demonstrated, clinical outcomes were similar between patients with and without respiratory copathogens [116].

High viral load, high-dose steroids, and myeloablative conditioning were found to be associated with prolonged viral shedding in HSCT recipients infected with HCoV [117], while a study comparing immunocompetent and immunocompromised children infected with HCoV identified, in multivariable models, younger age, underlying pulmonary disorders, respiratory copathogens (especially RSV), and an immunocompromised state as risk factors for LRTI [118].

Human bocavirus (HBoV), a small, non-enveloped, single-stranded DNA member of the Parvoviridae family, was identified by a Swedish group in 2005 [119], HBoV1 thus being the first virus to be discovered by a molecular virus screening; three additional species were subsequently discovered and added to the genus [120,121,122]; named HBoV2, HBoV3, and HBoV4, these species were largely found to be enteric pathogens, while HBoV1 has been associated with 2–19% of all upper and lower respiratory tract conditions throughout the globe [123,124,125]. In a study comparing HIV-infected with HIV-uninfected children with LRTI, HBoV was found in 9.5% of samples from the former and 13.3% of samples from the latter category; in both cases, the rate of coinfection with at least one other respiratory virus was high (69.4% and 74.4%, respectively) [126].

HBoV infections have been detected in patients with hematological malignancies or HSCT, though the extent of its morbidity and mortality are difficult to accurately ascertain due to high rates of copathogen detection and NAT identification of HBoV in asymptomatic patients [127,128]. One prospective study evaluating children with cancer diagnosed with viral respiratory infections identified HBoV in 8% of all RTIs where an etiological agent was found and 19% of diagnosed LRTIs, 57.1% of which had co-infections with other viruses [90]. The Pinana et al. group from Spain found HBoV infection in 6/79 HSCT recipients, 1 of which progressed to LRTI, with 0 fatalities, but with viral copathogens identified in 5/6 cases [113].

Human parainfluenza viruses (HPIV), medium size, negative-sense, single-stranded RNA member of the Paramyxoviridae family with five predominant identified serotypes, have long been known, together with RSV, to be respiratory pathogens primarily in children, causing mild URTID throughout the year and type-specific seasonal increases in URTID and LRTID (mainly laryngotracheitis, bronchiolitis, and pneumonia) during autumn and spring [129]. HPIV has been thoroughly researched, with epidemiological studies finding HPIV-1 and HPIV-3 to be responsible for up to one third of the more than 5 million LRTIs annually in the US in children younger than 5 years [130]; more so, of the 500,000 to 800,000 hospital admission for LRTID annually in persons younger than 18 years, approximately 12% have been found to be caused by HPIV-1, HPIV-2, and HPIV-3 [130]. As for other CRVs, interest in HPIV infections in immunocompromised patients has gained momentum in recent decades and HPIV has been reported to cause 2% to 7% of symptomatic infections in adult and pediatric HSCT and leukemia patients, at least a third of which manifested as LRTID [15,49,131,132,133,134,135]. Although typically self-limiting in immunocompetent populations, fatal outcomes have been known to occur in patients with leukemia and HSCT, with mortality reaching 17% in patients with HPIV pneumonia [15]. Another study demonstrated a strong association between HPIV-3 pneumonia and mortality in HSCT patients, with overall mortality reaching 35% at 30 days and 75% at 180 days post-transplant; the same study identified viral, bacterial, or fungal copathogens in 53% of patients with HPIV-3 pneumonia [136].

Several risk factors for progression to LRTI and/or mortality in hematology patients have been identified. Srinivasan et al. showed that HPiV infection in the first 100 days post-HSCT, use of steroids, and absolute leukocyte count (ALC) <100 cells/μL at the onset of infection were associated with LRTI, while African-American ethnicity, LRTI, use of steroids, mechanical ventilation, and ALC <100 cells/μL at the onset of infection were associated with higher mortality [133]. Ustun et al. showed that early infection (<100 days after HSCT), corticosteroid therapy, and the presence of copathogens were associated with higher mortality [135]. Detection of HPiV in the lower respiratory tract (through bronchoalveolar lavage or lung biopsy) of patients with HPiV LRTI was shown to be associated with worse outcome than detection of HPiV in the upper respiratory tract; the same study showed, in multivariable analysis, that oxygen requirement at diagnosis, low monocyte counts, and high-dose corticosteroid therapy (≥2 mg/kg/day) were also associated with high mortality [137] Either directly or by facilitating coinfection, it is clear that the impact of HPIV infections on mortality in at-risk populations is significant.

A brief summary of parts of the above data can be found in Table 4.

## 3. Management of CRV Infections

One of the key aspects in the proper management of CRV infections is the need for prompt and accurate identification of the respiratory pathogen. In this respect, great progress has been made in recent years, with multiplex PCR testing having gained significant popularity, being able to quickly screen for multiple pathogens [138,139].

In contrast with the rapid advancement in diagnostic techniques, treatment options for CRV infections remain limited. The development of potent antivirals varies greatly depending on the virus type. In the following paragraphs, we discuss the currently available and under treatment options for influenza, RSV, and rhinovirus infections.

### 3.1. Influenza

Rapid development of resistance to amantanes has made this class of antivirals obsolete [140], leaving neuraminidase inhibitors the only effective therapeutic agents against influenza infections for nearly two decades [141,142,143]. Concerns about potential emerging resistance to neuraminidase inhibitors have been addressed with the recent release of an endonuclease inhibitor, baloxavir marboxil, which has shown promising results in oseltamivir-resistant strains [144,145], but is still undergoing clinical trials to assess its efficacy when administered in association with neuraminidase inhibitors (NCT03684044), as well as safety, pharmacokinetics, and efficacy in pediatric patients (NCT03653364, ongoing, and NCT03629184, complete, no results available yet). Despite the promising initial results, concerns may arise regarding baloxavir marboxil’s low genetic barrier, which seem to have already lead to the emergence of antiviral resistance, with a reported rate of 7.9–9.7% among immunocompetent adults and adolescents [146]. Interestingly, the majority of patients in whom resistance occurred appear to have been infected with the A(H3N2) strain, whereas patients infected with the A(H1N1)pdm09 strain were shown, in a phase 2 study, to only develop resistance in 2.2% of cases [147], suggesting that the emergence of resistance may occur more frequently during the treatment of the A(H3N2) strain. Several other agents with activity against influenza viruses are currently under investigation, with compounds targeting components of the viral polymerase complex showing the most promise. The three potential targets are the PA protein (endonuclease, as targeted by the above-mentioned baloxavir), the PB1 protein (RNA-dependent RNA-polymerase), and the PB2 protein (cap-snatching subunit).

Favipiravir (T-705—Toyoma Chemical), a PB1 inhibitor, has shown broad activity against influenza virus, including neuraminase-inhibitor resistant strains, and has been approved for the treatment of pandemic flu in Japan. Furthermore, its activity extends to other RNA viruses as well, including those responsible for hemorrhagic fevers, making it an interesting target for further study and a potential therapeutic option for previously untreatable diseases [148]. Pimodivir (JNJ-63623872 or VX-787), a novel PB2 inhibitor, has been shown to be effective against influenza, demonstrating both virological (reducing viral shedding in nasal secretions) and clinical improvement [149,150]. Another PB2 inhibitor, CC-42344, is scheduled to begin preclinical trials in Q4 2020. Monoclonal antibodies targeting the hemagglutinin protein are another enticing avenue of research: MHAA4549A has shown promising results in phase 1 and 2a trials; VIS410 was shown to have favorable effects on symptom resolution and viral replication in adults with uncomplicated influenza A infections [151], while MHAA4549A (currently under development for the treatment of severe influenza infections) demonstrated dose-dependent antiviral activity in an influenza A virus challenge model (with the highest effect at a 3600 mg dose, a lower effect at a 400 mg dose, and, interestingly, no effect with a 1200 mg dose) [152]. Umifenovir, a broad-spectrum antiviral, is currently in use in Russia and China for the curative and prophylactic treatment of influenza infections, with some studies showing both experimental and clinical effectiveness [153,154].

### 3.2. Respiratory Syncytial Virus

One possible general antiviral agent investigated in uncontrolled trials has been aerosolized and/or systemic ribavirin, with or without intravenous immunoglobulin association. This has been proven to be safe to administer and appears to be effective in both preventing progression to LRTI and treatment of pneumonia caused by RSV and HPIV, although more randomized trials are needed to confirm this [52,155,156,157]. Concerns exist about the limitations of aerosolized ribavirin in regards to environmental exposure and potentially teratogenic effects in pregnant healthcare workers and visitors, special air-flow requirements, and high cost, making intravenous and oral administration viable alternatives [158,159].

Palivizumab, a monoclonal antibody targeting RSV protein F, has also emerged as a potentially effective agent, particularly in the prophylaxis of RSV pneumonia in both children and adults at increased risk of severe disease [160,161], but also in treatment of persistent RSV infection in children with leukemia [162] and improving outcomes in children with RSV LRTID [163].

Investigations of novel agents have been met with mixed results. ALS-008176 (lumicitabine), an oral nucleoside analogue prodrug with promising preliminary results [164], started several phase 2 trials studying its efficacy on RSV infections in children and infants (NCT03333317, NCT03332459), as well as one study following its effect on hospitalized adults with metapneumovirus infection (NCT03502694); these trials have, however, been suspended by the sponsor. GS-5806 (presatovir), a drug blocking RSV fusion protein [165], has undergone several trials, studying its effects on both general population (NCT02135614) and HSCT recipients with RSV URTI (NCT02254408) and LRTI (NCT02254421); results have not yet been published. AK0529 (ziresovir) [166], an orally bioavailable RSV fusion protein inhibitor, is currently undergoing a phase 2 study on adults with RSV infection in China (NCT03699202), while a multicentric study on a pediatric population has been completed, with results upcoming (NCT02654171). ALX-0171, a trimeric nanobody that binds to the antigenic site II of the RSV F protein with high affinity, has proven superior in vitro neutralization to that of palivizumab. [167]. Several other agents, including L-protein and N-protein inhibitors, the full description of which is beyond the scope of this review, are currently under study and have been described elsewhere.

### 3.3. Rhinovirus

Several treatment options have been explored for rhinoviral infections throughout the years. Capsid binders, capable of inhibiting viral entry, were considered to be promising agents, showing solid antiviral activity in vitro. A randomized trial, however, showed limited clinical benefit of intranasal pirodavir for naturally occurring rhinovirus colds; it did, however, reduce viral shedding on Days 3 and 7 [168], a benefit which may be of interest for patients with underlying haematological malignancies. Likewise, pleconaril, whether in oral or intranasal formulations, proved to only provide a modest clinical benefit, reducing the duration of symptoms in otherwise healthy adults with rhinoviral cold within 1 to 1.5 days [169]; it has also been proven to be potentially beneficial in neonates with severe enterovirus infection, warranting further research [170]. Oral vapendavir, a newer capsid binder, recently failed to prove superior to placebo in improving lung function or reducing asthma exacerbations in asthmatic patients; it did, however, result in a statistically significant antiviral effect (demonstrated by a negative RV PCR) when administered within 24 h of symptom onset (NCT02367313). Based on this finding, a phase 2 trial studying vapendavir treatment for HSCT patients with RV URTI was planned (NCT03024177), but was later withdrawn. Other classes of antiviral agents, including the 3C protease inhibitor rupintrivir, the nucleoside analog inhibitor MK-0608, and the phosphatidylinositol 4-kinase IIIβ (PI4K-IIIβ) kinase inhibitor PIK93, were shown to exhibit in vitro activity against Rhinovirus type C in a comprehensive study utilizing subgenomic-replicon- and infectious-virus-replication-based assays [171]. While clinical benefit for any of these agents has yet to be demonstrated, HSCT recipients/candidates would certainly stand to gain much from the judicious implementation of one such treatment option.

Regardless of treatment options, the ease with which respiratory viruses are transmitted makes the enforcement of infection control measures critical to reducing the spread of these infections, and doubly important in at-risk population groups [172]. Nosocomial transmission is also common, often with devastating consequences, making the need for adequate prevention all the more stringent [173]. In addition, deferral of HSCT has been shown to reduce the risk of progression to pneumonia [174].

## 4. Current Evidence-Based Guidelines for the Management of CRV Infections in Hematology Patients

The ECIL-4 group, after a thorough review of the literature, made several recommendations for the management of CRV infections in hematology patients [8] (Table 5).

The two non-influenza CRV infections for which potentially effective treatment recommendations can be made are RSV and HPIV, both of which benefit from aerosolized or systemic ribavirin and intravenous immunoglobulin; care should be taken in the case of aerosolized ribavirin to minimize exposure risk for medical staff. Deferral of therapy, whether HSCT or chemotherapy, appears to be a viable option in patients with CRV respiratory infections, though decisions should be made on a case-by-case basis.

The Guidelines for Preventing Infectious Complications among Hematopoietic Cell Transplant Recipients, cosponsored by the Center for International Blood and Marrow Transplant Research (CIBMTR), the National Marrow Donor Program (NMDP), the European Blood and Marrow Transplant Group (EBMT), the American Society of Blood and Marrow Transplantation (ASBMT), the Canadian Blood and Marrow Transplant Group (CBMTG), the Infectious Disease Society of America (IDSA), the Society for Healthcare Epidemiology of America (SHEA), the Association of Medical Microbiology and Infectious Diseases Canada (AMMI), and the CDC and updated in 2009, makes several in-depth, point-by-point recommendations, from general infection control acts (such as droplet and standard precaution) to specific measures (such as targeted therapy with ribavirin or palivizumab in RSV infection or vaccination or neuraminidase treatment in influenza infection). A summary of these recommendations can be found in Table 6.

## 5. Conclusions

Sheer abundance alone is enough to make the burden of CRV infections on healthcare systems throughout the world easily apparent, and, while the vast majority of them end up being self-limiting URTI diseases, their impact on global morbidity and mortality is not negligible. Hematological patients, by definition an at-risk population, make up a lot of those numbers insofar as mortality is concerned, with potentially fatal outcomes having been reported for the most benign of CRVs. While technological leaps in recent decades have greatly improved our knowledge of these viruses and facilitated diagnosis, long-term solutions, such as consistently effective treatment options, supported by large-scale studies, and adequate prophylaxis measures, through vaccination, continue to elude us. The ECIL-4 and CIBMTR, NMDP, EBMT, ASBMT, CBMTG, IDSA, SHEA, AMMI, and CDC guidelines are examples of progress made in both prevention and treatment, while the many therapeutic agents currently under investigation, despite not yet providing a definitive solution to an increasingly glaring problem, do represent first steps in finding remedies against microorganisms for which there previously had been none. In time, systematic management of CRV infections could prove very rewarding in terms of outcome improvement in hematology patients.

## Figures and Tables

**Table 1:** Epidemiology of some common respiratory viruses.

Study	Virus	Target Population	Results	Notes
Boivin et al., 2002 [1]	HMPV	General	2.3% (20/862 samples) over one winter season via viral culture	Also used RT-PCR to identify 38 previously unidentified respiratory viruses as HMPV, with ages <5 (35.1%) and >65 (45.9%) showing the highest prevalence
Nandhini et al., 2016 [3]	HMPV	General	5% (23/447 samples) over 2 years via RT-PCR	- 11/23 (48%) positive samples in age group 14–30 years, 22% and 4% in ages <5 and >51, respectively - 9/23 (39%) patients were admitted to the hospital and 26% needed mechanical ventilation
Arden et al., 2006 [4]	Multiple	General	315 specimens HRV: 44.4% HAdV: 6% HBoV: 4.8%	- Age group <5 years represented 78.9% of study population
Koskenvuo et al., 2008 [5]	HBoV	Pediatric ALL	125 samples/51 children; 7 samples (5.6%) positive for HBoV	- Age 0.4 to 15.3 years, mean age 5.9 years
Milano et al., 2010 [7]	HRV, HCoV	HSCT	215 patients HRV: 21% HCoV: 10%	- Study performed surveillance on HSCT recipients for 1 year after transplantation, with weekly samples collected during the first 100 days
Chemaly et al., 2012 [15]	HPiV	HSCT and leukemia	Incidence: - leukemia: 1% (80/7745) - autologous HSCT: 1.3% (23/1717) - allogenic HSCT: 5.5% (97/1756)	- AML (48%) and ALL (28%) most common malignancies - median time from HSCT to HPiV infection diagnosis: 70 days (range, 0–2836 days)
Spahr et al., 2018 [16]	RSV, HPiV, HMPV	HSCT	103 infections in 66 patients: 47% HPiV, 32% RSV and 21% HMPV	58.3% URTI, 35.9% LRTI, 5.8% unknown; overall mortality: 6%; infection episodes occurred >100 days post HSCT in 84.5% of cases
Wang et al., 2018 [17]	Flu, RSV, HPiV, HAdV	General	Flu: 5.7% RSV: 4.3% HPiV: 7.2% ADV: 1.6%	1300 patients enrolled over a 4 year surveillance period in Harbin, China

**Table 2:** ECIL-4 definitions of community-acquired viral respiratory tract infections [8].

Clinical Entity	Definition
Upper respiratory tract infection (URTI)	The detection of CRVs above and including the larynx (e.g., in samples from nose, pharynx, larynx, conjunctivae, or sinuses)
Upper respiratory tract infectious disease (URTID)	The detection of CRVs in upper respiratory tract fluid specimens, together with symptoms and/or signs and other causes excluded.
Lower respiratory tract infection (LRTI)	The detection of CRVs below the larynx (e.g., in samples from trachea, bronchus, bronchoalveolar sites)
Lower respiratory tract infectious disease (LRTID)	Pathological sputum production, hypoxia, or pulmonary infiltrates together with identification of CRVs in respiratory secretions, preferentially in samples taken from the sites of involvement

**Table 3:** Epidemiology of respiratory syncytial virus in hematology patients.

Study	Target Population	Results	Notes
Chemaly et al., 2006 [25]	Adult hematologic	107/343 (31%) of cases, over 2 years	- Progression to pneumonia in 68 cases, with 7 fatalities; 12 patients >65 years
Torres et al., 2007 [46]	Adult leukemia	52 patients with leukemia and RSV infection identified over 4 years 5 months	- 27 (52%) cases of pneumonia, 5 fatalities (10%), of which 4 received and 1 did not receive ribavirin-based treatment
Avetisyan et al., 2009 [47]	HSCT recipients	32/275 (11.6%) transplanted patients over an 8 year period	- 14/32 (43.75%) cases of pneumonia, 5 fatalities, of which 3 were assessed to have died from RSV LRTI (1.1% of all patients, 9.4% of patients with RSV infection)
Martino et al., 2005 [49]	HSCT recipients	386 patients, 177 samples; 19 (4.92%) tested positive for RSV over 4 years	- Also tested for HMPV (4.14%) and influenza A/B (10.1%)
Hassan et al., 2003 [50]	HSCT recipients	626 transplant recipients of which 27 patients with 29 (4.3%) episodes of any viral RTI, 8 (27.58%) of which were RSV	- Other viruses (no. cases): HRV (11), Influenza A (5), HPiV3 (4), HEnV (2), CMV (9) - RSV pneumonia in 2/8 cases, one death, not attributable to infection
McCarthy et al., 1999 [51]	HSCT recipients	336 transplant recipients, 26 (6.3%) RSV infections	- Ages 0.5–31.1 years, median age 10.6 years - 15 LRTIs with 5 deaths attributable to RSV infection (19.2% of RSV infections and 1.48% of all patients)

**Table 4:** Summary of epidemiological data.

Virus	General Population		Hematologic Malignancy/HSCT		Risk Factors
	Incidence	Mortality	Incidence	Mortality	
**RSV**	2005 estimates: 33.8 million episodes (22% of all LRTIs) in children <5 years old	2005 estimates: 66,000–199,000 in children <5 years old	0.3–14% (pediatric), 1–31% (adult)	32%	Host-related; ISI: neutropenia, lymphopenia, age <40 years, graft-versus-host disease, corticosteroid use, myeloablative chemotherapy, time from HSCT
HMPV	2–7%	Self-limiting	2.5–9%	6%; 27% in patients who develop HMPV LRTI	Host- and virus-related: prematurity, female sex, genotype B virus (immunocompetent children); hypoxia, nosocomial acquisition, hematologic malignancy (cancer patients)
HRV	52–80% of common colds	Self-limiting	23–62% of URTIDs, 65% of LRTIDs (children) 22.3% (adults)	6% (URTID), 41% (LRTID)	Low monocyte count, oxygen requirement at diagnosis, corticosteroid use ≥ 1 mg/kg
HCoV	10–30% of common colds	10.8% (SARS), 35.67% (MERS); otherwise self-limiting	11.1%	54% in patients with LRTI and require oxygen at diagnosis	High viral load, high-dose steroids and myeloablative conditioning (for prolonged viral shedding)
HBoV	2–19% of all RTIs	Self-limiting	8% of all RTIs with found etiology, 19% of diagnosed LRTIs	0% in found studies	Difficult to ascertain due to frequency of copathogens
HPiV	12% of 500,000–800,000 patients <18 years old admitted with LRTIDs	Typically self-limiting	2–7% of symptomatic RTIs, 1/3 of which manifest as LRTID	17–35% (and as high as 75%, with high frequency of coinfection)	With progression to LRTI: Temporal proximity to HSCT, steroid use, low ALC at onset With mortality: African-American ethnicity, low ALC, LRTI, steroid use, mechanical ventilation

**Table 5:** ECIL-4 recommendations for the management of community-acquired respiratory infections in hematology patients [8].

Condition	Recommendation	Strength of Recommendation/Quality of Evidence *
Patients planned for allogenic HSCT with CRV respiratory tract infectious disease (RTID)	Deferral of conditioning therapy should be considered	BII
Patients with hemato-oncological disease and CRV RTID	Deferral of conditioning/chemotherapy could be considered	BIII
Patients undergoing allogenic HSCT or HSCT recipients with RSV URTI and risk factors for progression to LRTID	Should be treated with aerosolized or systemic ribavirin and IVIG	BII
Allogenic HSCT recipients with HPiV LRTID	Aerosolized or systemic ribavirin and IVIG may be considered	BIII
Allogenic HSCT recipients with CRV RTID other than RSV and HPiV	Aerosolized or systemic ribavirin and IVIG cannot be recommended	CIII

* according to grading system used by Infectious Diseases Society of America.

**Table 6:** Guidelines for Preventing Infectious Complications among Hematopoietic Cell Transplant Recipients recommendations [175].

Condition	Recommendation	Strength of Recommendation/Quality of Evidence *
HSCT recipients with symptoms of RTID	Preventing exposure: strict infection control measures should be implemented and modified as needed once the etiology is identified	BIII
HSCT recipients with RSV URTI	Aerosolized ribavirin can be preemptively administered, especially in patients with lymphopenia (during the first 3 months after HSCT) and preexisting lung disease (late after HSCT)	CIII
Pediatric HSCT recipients at risk for primary RSV disease (<4 years old)	Monthly palivizumab prophylaxis can be administered during RSV season (November–April)	CIII
HPiV or HMPV infection	No recommendations can be made	Lack of data
HSCT recipients at highest risk for adenovirus infection (refractory graft versus host disease, umbilical cord blood transplantation, haploidentical transplantation, stem cell graft T cell depletion of >2–3 log_10_, use of anti-T cell antibodies)	Can been monitored weekly for active adenovirus infection by PCR for either the first 6 months after HSCT or for the duration of severe immunosuppression/lymphopenia	CII
HSCT recipients with adenovirus infection	If possible, rapid tapering or withdrawal of immunosuppression constitutes the best way to prevent progression of infection	AII
All HSCT candidates and recipients	Lifelong seasonal influenza vaccination with the trivalent inactivated vaccine *	AII
Patients with influenza infection and potential HSCT recipient/candidate contact	Should be placed droplet and standard precautions to prevent transmission	AIII
HSCT recipients <6 months after HSCT, during community influenza outbreaks that lead to nosocomial outbreaks	Should receive prophylaxis with neuraminidase inhibitors	AII
HSCT patients with influenza URTI	Should receive early preemptive therapy with drugs to which the circulating strain is known to be susceptible	AII

* updated guidelines routinely recommending quadrivalent vaccine were not found.

## Abbreviations

HMPV	human metapneumovirus
HRV	human rhinoviruses
HAdV	human adenoviruses
HBoV	human bocavirus
HCoV	human coronavirus
HPiV	human parainfluenza virus
AML	acute myeloid leukemia
ALL	acute lymphoblastic leukemia
HSCT	hematopoetic stem cell transplant
NPA	nasopharyngeal aspirate
RSV	respiratory syncytial virus
HSCT	hematopoetic stem cell transplant
LRTI	lower respiratory tract infection
HRV	human rhinoviruses
HPiV	human parainfluenza virus
HEnV	human enteroviruses
CMV	cytomegalovirus
RSV	respiratory syncytial virus
HMPV	human metapneumovirus
HRV	human rhinovirus
HCoV	human coronavirus
HBoV	human bocavirus
HPiV	human parainfluenza virus
U/L/RTID	upper/lower/respiratory tract infectious disease
SARS	severe respiratory syndrome
MERS	Middle East respiratory syndrome
ISI	immunodeficiency scoring index
ALC	absolute leukocyte count
